# Trefoil Factor Family Member 2 (TFF2) as an Inflammatory-Induced and Anti-Inflammatory Tissue Repair Factor

**DOI:** 10.3390/ani10091646

**Published:** 2020-09-14

**Authors:** Abdelaziz Ghanemi, Mayumi Yoshioka, Jonny St-Amand

**Affiliations:** 1 Department of Molecular Medicine, Faculty of Medicine, Laval University, Québec, QC G1V 0A6, Canada; abdelaziz.Ghanemi@crchudequebec.ulaval.ca; 2 Endocrinology and Nephrology Axis, Functional Genomics Laboratory, CHU de Québec-Université Laval Research Center, Québec, QC G1V 4G2, Canada; mayumi.yoshioka@crchudequebec.ulaval.ca

**Keywords:** trefoil factor family member 2 (TFF2), inflammation, tissue repair

## Abstract

Trefoil factor family member 2 (TFF2) is known for its involvement in mucosal repair. Whereas it is overexpressed during inflammatory processes, adding TFF2 leads to an anti-inflammatory effect that would contribute to create the microenvironment required for tissue repair. These properties present TFF2 with a homeostatic pattern during inflammatory processes as illustrated by selected examples.

## Dear Editor,

Compared to the diverse physiological entities, digestive and respiratory systems represent the tissues that interact the most with exogenous organisms and molecules, as they represent the two “entrances” of the body. This anatomical property exposes these systems to diverse stimuli and injuries leading to inflammatory reactions, especially with their rich blood flow and close interactions with the immune system. In addition, their mucosa have a relatively high regenerative and repair activity. Within the context of mucosal repair, trefoil factor family member 2 (TFF2), also known as spasmolytic polypeptide and isolated in 1982 [1], is a biological factor known for its involvement in mucosal repair, protection and proliferation especially within both digestive and respiratory systems [2,3,4,5,6,7,8]. TFF2 represents an important component and a stabilizer of the gastric mucus with the property of binding to the mucin MUC6 [9] and is also involved in tissue remodeling [2,10]. It is expressed in different species such as mouse [11], cow [12], rat [13], pork [9] and human [14]. In veterinary science, the animal models of TFF2-modified expression illustrate the importance of this protein in animal health as shown by studies investigating obesity, gastric secretion, asthma, etc [2,3,10,11].

These TFF2 properties are reflected by the increased susceptibility to injury seen in TFF2-deficient mice. Indeed, TFF2-deficient mice have an increased gastric ulceration degree compared to wild-type mice following indomethacin administration [3]. Since there is numerous inflammatory diseases [4,15,16,17] that develop in the digestive and respiratory systems, we would like to summarize hypothetic links between the TFF2 and selected inflammatory-related processes [2,18,19,20].

TFF2 has been shown to be overexpressed (or upregulated) following inflammations or inflammatory conditions [18] such as in asthma [2], gastrointestinal ulcerative disease [19] and allergic airway inflammation [20]. Furthermore, knowing that some interleukins (IL) have been linked to tissue repair [21,22,23], such regulation could also be under the control of selected cytokines since, for instance, IL-4 and IL-13 induce TFF2 in the lung [20]. Other treatments, also leading to cell damage, upregulate TFF2 or *TFF2* expression, such as hypoxia [24] and aspirin in which the damages are also associated with hypoxia [24,25]. This suggests that the upregulation would be a response of the inflammation-induced damage rather than the inflammation itself, which correlates with aspirin damage-induced activation of *Tff2* gene in rats [13]. This would mean that TFF2 would not be required to develop the inflammation but would rather increase with inflammation, either induced by inflammation or the factor triggering the inflammation. This TFF2 induction would initiate the healing and repairing process that counteracts the inflammation-induced damage, which could be a protective mechanism such as during chronic superficial gastritis [26].

Interestingly, other studies have pointed TFF2 with a potential anti-inflammatory effect. For instance, a recombinant human TFF2 was shown to reduce colitis inflammation in a rat model; it increases the colonic epithelial repair rate [27]. Within the same line, applying TFF2 does reduce inflammatory indexes in a hapten colitis rodent model and has even been suggested as a therapeutic scaffold for inflammatory bowel disease treatment [28]. Importantly, TFF2 treatment reduces fibrosis (subepithelial collagen deposition) in a murine model of chronic allergic airways disease [2], which could indicate a reduced fibrogenesis in tissues undergoing inflammation [29,30]. Thus, TFF2 effects are not limited to an anti-inflammatory effect but would also reduce the tissue fibrosis. Both effects are towards tissue repair and counteract the deteriorating inflammatory consequences (damage and fibrosis) as well. This could explain the TFF2 beneficial effect on intestinal inflammation in animal models, which would involve reducing both macrophage responsiveness [28] and leukocyte recruitment [31], regulating the NO-mediated inflammation (monocyte) [32] and blocking inflammatory cell recruitment [28] within its mechanism. On the same path, TFF2 is also expressed during gastric cancer [33,34]. This could indicate that the presence of TFF2 aims to limit the cancer-induced inflammatory damages. It could also represent an attempt to limit cancer growth as suggested by an in vitro study that shows the inhibition of the growth of gastric cancer cells by TFF2 expression [35].

It is worth precising that the anti-inflammatory effect or fibrosis reduction have been observed when exogenous TFF2 was added in different conditions [2,27,32] rather than when the inflammation-related endogenous TFF2 was overexpressed (since inflammation develops although the inflammation-induced upregulation of TFF2 expression [2,18,19,20]). This highlights TFF2 overexpression as an attempt to limit the inflammation and its consequences (such as fibrogenesis). Such an anti-inflammatory effect or fibrosis reduction would be among the main mechanisms underlying the pathways via which TFF2 mediates its mucosal protection. Although the inflammatory-induced TFF2 overexpression (not its exogenous addition) would not lead to a measurable effect on inflammation or fibrosis, inflammatory-related TFF2 expression would probably contribute to create the biological environment required for tissue repair, but not only through recruiting selected factors and interacting with biomolecules such as mucins [18,36].

Interestingly, probiotics have been shown to increase the production of TFF2 in the mouse stomach [37]. Probiotics also have, in addition to roles in tissue repair [38], anti-inflammatory effects especially in the intestine [39], which is one of the key tissues of TFF2 expression. Thus, TFF2 might be among the pathways linking probiotics to the anti-inflammatory and tissue repair effects, probably involving immunological mechanisms impacted by probiotics [40]. In addition, the reported antibiotic activity of TFF2 [14] could be complimentary in both inflammation and immunological regulation towards reducing inflammation-related damages.

*Tff2* has been recently characterized as a high-fat diet-induced gene in the intestinal mucosa [41] and the knock out of this gene lead to a protection from high-fat diet-induced obesity [11,42]. Both these facts could be further considered for the future exploration of the links between inflammation and metabolics. Indeed, obesity, for which a high-fat diet increases its development, also represents a risk factor for both inflammation and cancer development. Therefore, the metabolic implications of TFF2 could be behind a part of the inflammatory and cancer processes, especially based on known links between metabolic activities and the factors related to inflammation and cancer [43,44,45]. Within this context, IL could complete TFF2 roles during tissue repair. For instance, tissue injuries induce IL-6 production [46], which is required for gastric homeostasis [47] and has been shown to play metabolic roles [43]. This would indicate complementarity roles between TFF2 and IL during tissue repair by contributing to create the microenvironment as well as the metabolic conditions required for post-injury repair and counteracting the tissue damages. Within the context of diet, it is also worth mentioning that a diet rich in antioxidants would have a beneficial effect on inflammation development [48]. Moreover, the high-fat diet-induced *Tff2* gene expression could be related to counteracting inflammation damages, since high-fat diet induces oxidative stress [49] and is usually associated with obesity [50,51] which has both oxidative stress [52] and inflammation [53,54] in its context.

Moreover, the other TFFs (TFF1 and TFF3) would require additional exploration within the context of inflammation because of their implication in the inflammation process [4] as well as the possible expression interdependence linking TFF2, TFF1 and TFF3 [55,56]. Furthermore, the inflammatory properties of TFFs correlate with their immunological roles [28]. This could also justify the expression of TFFs (minute amounts) in the immune and central nervous systems [57] as well as in cancers [12,58] as regulatory factors. Deeper understudying of TFF2 implications in inflammation or inflammatory-related diseases and conditions would allow developing new methods to confirm diagnosis, make prognosis or follow a therapy efficiency based on TFF2 expression variation as a biological marker, such as in tumors [33]. Moreover, these implications of TFF2 in inflammation would suggest the potential usage of TFF2 or targeting TFF2-related pathways to develop novel therapies or optimize those in usage for diseases and conditions involving an inflammatory component. The “homeostatic property” of TFF2 exposed is similar to the one we reported for the secreted protein acidic and rich in cysteine (SPARC) during inflammation [59] and cancer [60]. Interestingly, SPARC is also involved in response to injury and tissue remodeling [61,62]. Such opposing effects may broaden the application horizons and these two examples of TFF2 and SPARC illustrate mechanistic links between the need to control inflammation as well as adapting cellular patterns (metabolism, structural shape, etc.) during tissue repair and regeneration processes. Elucidating these links will expand therapeutic perspectives based on molecular pathways of diseases in animals and humans.
